# Contrast-Enhanced Spectral Mammography Assessment of Patients Treated with Neoadjuvant Chemotherapy for Breast Cancer

**DOI:** 10.3390/curroncol28050298

**Published:** 2021-09-06

**Authors:** Katarzyna Steinhof-Radwańska, Anna Grażyńska, Andrzej Lorek, Iwona Gisterek, Anna Barczyk-Gutowska, Agnieszka Bobola, Karolina Okas, Zuzanna Lelek, Irmina Morawska, Jakub Potoczny, Paweł Niemiec, Karol Szyluk

**Affiliations:** 1 Department of Radiology and Nuclear Medicine, Prof. Kornel Gibiński Independent Public Central Clinical Hospital, Medical University of Silesia, Medyków 18, 40-514 Katowice, Poland; abarczykgutkowska@gmail.com; 2 Students’ Scientific Society, Department of Radiology and Nuclear Medicine, Medical University of Silesia, Medyków 18, 40-514 Katowice, Poland; grazynska.anna@gmail.com (A.G.); karolina.okas@o2.pl (K.O.); zuzkalelek@gmail.com (Z.L.); irmina.morawska@gmail.com (I.M.); potocznyjakub123@gmail.com (J.P.); 3 Department of Oncological Surgery, Prof. Kornel Gibiński Independent Public Central Clinical Hospital, Medical University of Silesia, Medyków 18, 40-514 Katowice, Poland; ajlorek@O2.pl; 4 Department of Oncology and Radiotherapy, Prof. Kornel Gibiński Independent Public Central Clinical Hospital, Medical University of Silesia, Medyków 18, 40-514 Katowice, Poland; igisterek@sum.edu.pl (I.G.); bobola.agnieszka@gmail.com (A.B.); 5 Department of Biochemistry and Medical Genetics, School of Health Sciences in Katowice, Medical University of Silesia, Medyków 18, 40-752 Katowice, Poland; pniemiec@sum.edu.pl; 6 1st Department of Orthopaedic and Trauma Surgery, District Hospital of Orthopaedics and Trauma Surgery, Bytomska 62, 41-940 Piekary Śląskie, Poland; szyluk@urazowka.piekary.pl

**Keywords:** contrast-enhanced spectral mammography, breast cancer, neoadjuvant chemotherapy, complete response, response evaluation criteria in solid tumors

## Abstract

Background: Evaluating the tumor response to neoadjuvant chemotherapy is key to planning further therapy of breast cancer. Our study aimed to evaluate the effectiveness of low-energy and subtraction contrast-enhanced spectral mammography (CESM) images in the detection of complete response (CR) for neoadjuvant chemotherapy (NAC) in breast cancer. Methods: A total of 63 female patients were qualified for our retrospective analysis. Low-energy and subtraction CESM images just before the beginning of NAC and as a follow-up examination 2 weeks before the end of chemotherapy were compared with one another and assessed for compliance with the postoperative histopathological examination (HP). The response to preoperative chemotherapy was evaluated based on the RECIST 1.1 criteria (Response Evaluation Criteria in Solid Tumors). Results: Low-energy images tend to overestimate residual lesions (6.28 mm) and subtraction images tend to underestimate them (2.75 mm). The sensitivity of low-energy images in forecasting CR amounted to 33.33%, while the specificity was 92.86%. In the case of subtraction CESM, the sensitivity amounted to 85.71% and the specificity to 71.42%. Conclusions: CESM is characterized by high sensitivity in the assessment of CR after NAC. The use of only morphological assessment is insufficient. CESM correlates well with the size of residual lesions on histopathological examination but tends to underestimate the dimensions.

## 1. Introduction

Neoadjuvant chemotherapy (NAC) is currently one of the basic methods of treatment of locally advanced breast cancer [1,2]. The main advantage of NAC use is the possibility of reducing the mass of previously inoperable tumors to allow surgical intervention. In the case of massive yet operable tumors, NAC provides a decrease of their mass to a size that makes conservative surgical treatment possible. Thus, a better cosmetic effect will be achieved and fewer post-operative complications will be present [3,4,5]. Besides reducing the tumor mass and enabling thereby offering better conditions for local treatment (breast-conserving therapy (BCT)), NAC provides unique opportunities for in vivo chemotherapeutic evaluation of cancer cells’ sensitivity, as well as for the search for new biomarkers of therapeutic response. In addition to locally advanced breast cancers, NAC has applications in some molecular subtypes of breast cancer. It is recommended for the following kinds of cancer patients: hormone receptor positive/human epidermal growth factor receptor 2 negative (HR+/HER2-) low and high risk, human epidermal growth factor receptor 2 positive (HER2+) and triple negative breast cancer (TNBC) of any size and stage. NAC is also a method of choice in cases of inflammatory breast cancer (IBC) [6,7,8].

Consequently, in the event of poor response and progression of the disease, NAC offers a chance to alter the treatment scheme or refer a specific patient for surgical treatment. Apart from the predictive and prognostic factors known to date, including staging, grading, HER2 status, hormone receptors status, and Ki-67% index, the therapeutic response of the tumor to NAC provides information about the patient’s prognosis [9,10,11]. Achieving complete response (CR) upon completion of neoadjuvant therapy and surgical resection is associated with improved survival rates. CR was approved by the US Food and Drug Administration (FDA) in 2020 as the final point of clinical studies in the neoadjuvant therapy of early breast cancer with high recurrence risk [12]. However, it has to be mentioned that NAC response parameters can differ among patients due to the internal heterogeneity of tumors caused by molecular features, their clinical status, and morphological appearance. It is estimated that approximately 10–35% of female patients diagnosed with breast cancer remain chemotherapy-resistant, while in 5% NAC is completely non-reactive and leads to the disease progression [13]. In cases of such patients, NAC is a method of much lower efficacy: it deteriorates prognosis, delays surgical intervention, and increases the overall cost of therapy. Most recent research indicates its unsatisfactory results in decreasing or removing metastatic sites [11,14,15]. Despite the limitations described above, NAC is widely used in breast cancer therapy. It is estimated that approximately 7–27% of female patients suffering from breast cancer in Europe, the USA, and Australia undergo NAC treatment [2].

Evaluating the tumor response to NAC is key to planning further therapy. Underestimation of residual lesions after NAC can lead to incomplete tumor resection during surgery. This can lead to relapse or a need for reoperation. On the other hand, overestimation may result in overly radical surgical intervention, which can lead to much more serious complications. The gold standard of NAC response analysis is still histopathological examination. However, such examinations are conducted postoperatively. Due to this, it is crucial to determine and find reliable, non-invasive, and effective imaging diagnostic methods to assess NAC response in tumors.

The diagnostic imaging techniques currently used to assess response to NAC are conventional mammography (MG), ultrasound (US), photon emission tomography, and magnetic resonance imaging (MRI) with contrast enhancement (CE-MRI). CE-MRI is considered to be the most effective of these methods [16,17]. Multiple studies have shown that dynamic contrast-enhanced MRI is the optimal imaging tool to determine disease response, with a sensitivity reaching 90%, specificity of 60% to 100%, and accuracy of approximately 91%. However, CE-MRI remains an expensive method with limited access in local diagnostic centers. Patients with contraindications and who are unable to tolerate examination conditions (i.e., claustrophobia, psychic comfort, old-type pacemakers) cannot undergo such examinations [18,19,20,21,22]. Furthermore, some research shows that MRI may under- as well as overestimate the size of residual lesions in 18% of cases [23]. Considering all these aspects, the search for new, more efficient, and potent diagnostic methods is still important.

Contrast-enhanced spectral mammography (CESM) is a novel technique intensively developed in the last few years. This diagnostic method was accepted by the FDA for clinical use in the United States in 2011. During CESM examination, approximately 2 min after contrast administration (chelated iodine-based X-ray contrast agent), dual-energy mammography is performed. During one examination, two images are acquired: one resembling conventional mammography (low-energy images of equal or noninferior quality to those of standard digital mammography) and another—the subtraction image—demonstrating areas of increased contrast enhancement (like in the case of the CE-MRI that uses Gadolinium-based contrast dose) [24,25]. Another advantage of CESM is that it provides both the anatomical and morphological characteristics of the lesions in the breast and regions of increased neoangiogenesis. CESM is characterized by comparable or even better sensitivity and a higher negative predictive value (NPV) and positive predictive value (PPV). Moreover, in contrast to MRI, CESM allows the visualization of microcalcifications [26,27]. Due to the much shorter examination time, lower costs, higher comfort, and low anxiety involved, patients tend to cooperate more fully and prefer CESM to MRI [28].

Consequently, the use of CESM in the evaluation of response to NAC is justified, bearing in mind the efficiency of this method, which is comparable to CE-MRI. According to the European Society of Breast Cancer (EUSOBI) recommendation in 2017, CESM can be considered as an alternative to contrast-enhanced MRI in the case of contraindications to MRI. CESM is a younger and less-studied technique than MRI. In CESM, there is an extra radiation dose of approximately 20%, but both images—low- and high-energy—still imply an X-ray dose below the recommended dose for mammography. In patients treated with neoadjuvant chemotherapy for breast cancer when a CESM examination is planned, additional MG can be avoided, with the possibility of saving the radiation dose [29].

Therefore, our study aimed to evaluate the effectiveness of low-energy and subtraction CESM images in the detection of CR for neoadjuvant chemotherapy in breast cancer. In addition, we decided to correlate the residual changes assessed with CESM low-energy and subtraction images.

## 2. Materials and Methods

A total of 63 female patients (mean age of 53.32 ± 9.47 years; ages ranged from 33 to 75 years) were qualified for our retrospective analysis. Between 2016 and 2019 they were put forward for NAC due to breast cancer in the University Clinical Center of the Medical University of Silesia in Katowice, Poland. Inclusion criteria encompassed: a diagnosis of breast cancer (based on core needle biopsy), minimal stage of T1N+, age of more than 18 years, completion of NAC treatment, surgery after NAC, and a complete set of imaging examinations—CESM; ultrasonography (US) of the breast, lymph nodes, and abdomen; and chest X-ray. Exclusion criteria were as follows: known BRCA1 or BRCA2 mutation (to provide the highest radioprotection), pregnancy, anaphylaxis or anaphylactoid reaction to a contrast agent in medical history, e-GFR of less than 60 mL/min, chronic kidney disease in stage 3 or higher, and incomplete NAC. Patients with significant post-biopsy changes (e.g., hemorrhage) affecting image quality were also excluded. A full description of the study group is in Table 1.

Due to the retrospective nature of this study, the local ethics committee of the Medical University of Silesia repealed the requirement for informed consent (decision number PCN/0022/KB/157/20). All the test procedures were carried out in compliance with the ethical principles of the 1964 Helsinki Declaration and its subsequent amendments.

### 2.1. Neoadjuvant Systemic Therapy

After the end of breast cancer diagnosis, each patient’s final decision concerning the proper treatment was made by the multidisciplinary breast cancer team (BCU) unit, taking into account patients’ preferences. Following the decision by the BCU team, 98% of patients received chemotherapy based on anthracyclines and taxanes (for a period of 12 weeks), including 30% in the “dose-dense” regimen. Only one patient did not receive anthracyclines due to a limited dose of anthracyclines in earlier treatment of Hodgkin’s disease. Due to the lack of public funding for trastuzumab in this center in the years 2016–2019 (no drug prescription government program), all the cancer patients with positive HER2 were treated outside our center and were not included in this analysis.

### 2.2. CESM Examination

All CESM examinations were performed in our hospital. They were carried out with the use of a digital mammography device dedicated to performing dual-energy CESM acquisitions (SenoBright, GE Healthcare, 3000 N. Grandview Blvd., Waukesha, WI, USA). An intravenous injection of 1.5 mL/kg of body mass of non-ionic contrast agent was performed using a power injector at a rate of 3 mL/s with a bolus chaser of 30 mL of saline. In CESM mode, the device automatically performed a pair of exposures (low- and high-energy) in each view. Specific image processing of low-energy and high-energy images was done. This processing aimed to obtain subtraction images to highlight contrast enhancement and suppress structured noise due to fibroglandular breast tissue. The morphological information obtained from low-energy images of CESM is similar to the morphological information given by standard mammography and the functional information obtained from subtraction images of CESM visualizes the vascularization of breast lesions [24,25,30]. For each view, the CESM technique made it possible to obtain two images: a low-energy acquisition at 26–30 kVp and a high-energy acquisition at 45–49 kVp, with these values depending on breast density and thickness. Motion blur could be sometimes observed on subtraction images due to movements between the acquisition of low- and high-energy images. All of the images obtained were in the DICOM format [31]. The total examination time was usually 10 min. After examination, the patients were observed for approximately 30 min for any adverse reactions that may have occurred after administration of the contrast agent.

### 2.3. Imaging Interpretation

All CESM examinations were carried out two times: just before the beginning of NAC and as a follow-up examination 2 weeks before the end of chemotherapy to evaluate its effect (and to inform decisions about possible changes in therapeutic strategies). For our retrospective analysis, we performed renewed CESM image evaluations with every patient. Such evaluations were made separately by two different radiologists with 20 years of experience and a minimum of 5 years’ experience in CESM image interpretation. Radiologists were blinded from each other and from the results of histopathological examination. CESM images were assessed according to the Breast Imaging-Reporting and Data System (BI-RADS) scale. Three measurements were taken in the CC and MLO projections, while the statistical analysis encompassed one—i.e., the largest dimension of the tumor. The tumor dimensions were compared while analyzing:

- Low-energy images from two consecutive contrast-enhanced spectral mammograms (taken before the start and at completion of neoadjuvant chemotherapy);
- Subtraction images from two consecutive contrast-enhanced spectral mammograms (taken before the start and at completion of neoadjuvant chemotherapy).

The response to preoperative chemotherapy was evaluated based on the Response Evaluation Criteria in Solid Tumors (RECIST) 1.1 criteria. The response was classified as follows: complete response (CR, disappearance of all lesions); partial response (PR, ≥30% dimensional reduction), stable disease (SD, <30% dimensional reduction/<20% dimensional increase), and progressive disease (PD, ≥20% dimensional increase) [17,32]. For this article, PR, SD, and PD have been classified as “non-CR”.

After surgery, a comparison of the NAC response, evaluated with the use of CESM (both low-energy and spectral images), to the histopathological study results was undertaken. Our analysis of histopathology (HP) was used as a “golden standard” against which the effectiveness of both CESM image types in NAC response detection could be analyzed.

### 2.4. Histopathological Examination

The histopathological examination was conducted in the Histopathology Laboratory of our center by two pathologists with broad experience in breast cancer diagnostics. The greatest dimension of the tumor, necessary for determining the T descriptor in the pTNM classification apart from the macroscopic measurement, was verified histopathologically. This verification was undertaken using a microscope and the Olympus Cell Sens Dimension^®^ software (Japan, 2013). Tumors up to 2 cm were excised whole, serially, on a cross-sectional basis with a margin of 0.2 to 0.4 cm and embedded in a paraffin block after each cross-section. Tumors measuring over 2 cm that could not fit within a single paraffin block were divided into two or more parts by making parallel cuts of the lesion. Next, they were marked in pairs with ink of the same color and the individual layers were given numbers to allow for the restoration of the largest section of the tumor. The T value of the tumor was the total of the parallel measurements of the particular parts of the lesion.

### 2.5. Statistical Analysis

Tumors that underwent histopathology examination were used as the “gold standard” and compared to the tumor sizes derived from CESM images. The normality of the distribution was assessed using the Shapiro–Wilk test and the continuous variables were summarized using the arithmetic mean with standard deviation for data following a normal distribution or a median with quartiles 1 and 3 for data demonstrating anon-normal distribution. Comparison of four respective aspects referring to the maximal tumor dimension (defined as the maximum of three dimensions measured in the CESM and histopathology) included the Pearson’s correlation coefficient (R-value) in order to measure the strength of the relationship between low-energy and subtraction images of CESM measurements. Paired *t*-tests were used to assess mean differences between each analyzed study participant. The correlations of data are illustrated by plotting the actual measurements while all paired measurements for each patient are summarized using paired linear plots. A *p*-value of <0.05 was considered statistically significant. The diagnostic performance indexes for low-energy and subtraction images for complete response and non-complete response were tested using the Clopper–Pearson test with 95% confidence intervals. Statistical analyses were carried out using Statistica 10 (StatSoft Inc., Tulsa, OK, USA) and MedCalc Statistical Software 16.4.3 (MedCalc Software by, Ostend, Belgium) software.

## 3. Results

Before neoadjuvant chemotherapy, the average size of the tumors varied from 34.4 mm for low-energy CESM to 34.3 mm for CESM subtraction images. After neoadjuvant chemotherapy, their average sizeswere17.6 mm for low-energy images and 8.5 mm for CESM subtraction images. The average size of the lesions in the histopathological examination was 11.1 mm (Table 2). The average reduction of the tumors reached 52.22% of the initial tumor mass based on low-energy images, and this even reached 78.76% in the case of CESM subtraction images.

**Figure 1:**
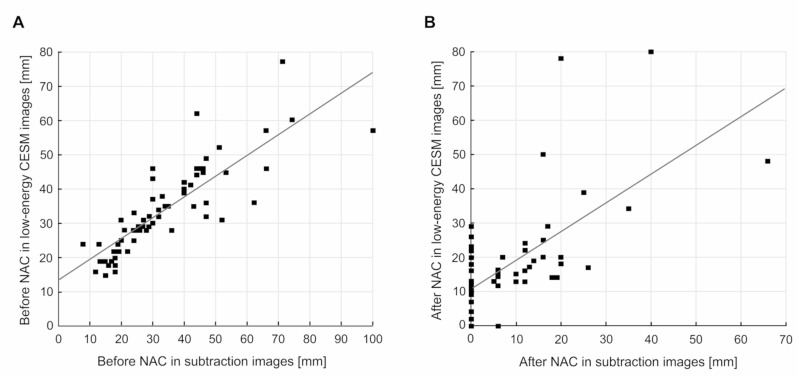
Correlations between: (**A**) the maximum tumor size before NAC in low-energy and subtraction CESM images: R = 0.89, *p* < 0.01; (**B**) the maximum tumor size after NAC in low-energy and subtraction CESM images: R = 0.57, *p* < 0.01.

When comparing the maximum tumor dimensions before neoadjuvant chemotherapy for low-energy and subtraction CESM images, a high degree of correlation in the Spearman’s analysis (R = 0.89, *p* < 0.01) was noticed. When the comparison between the maximum tumor dimensions after neoadjuvant therapy for low-energy and subtraction CESM images is considered, the correlation between the results can be described as moderate (R = 0.57, *p* < 0.01) (Figure 1).

**Figure 2:**
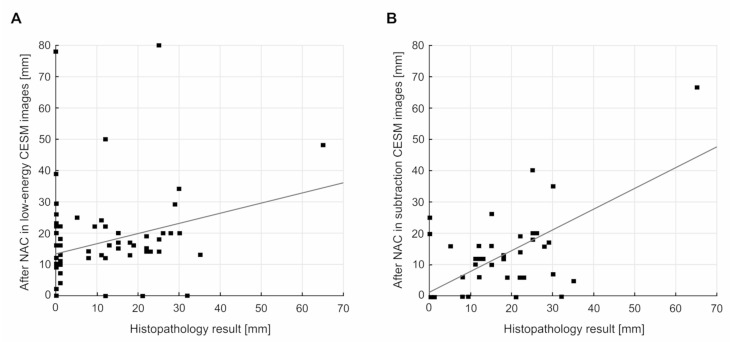
Correlations between: (**A**) the maximum tumor sizes after NAC in low-energy CESM images and in histopathology results: R = 0.26, *p* < 0.04; (**B**) the maximum tumor sizes after NAC in low-energy CESM images and in histopathology results: R = 0.67, *p* < 0.01.

A certain correlation, defined as moderate (R = 0.44, *p* < 0.01), can also be observed upon the comparison of the maximum tumor reductions for low-energy and subtraction CESM images. In terms of comparing the measurements of the maximum size for low-energy and subtraction CESM images following NAC and the maximum size in the histopathological examination, there was a low level of correlation for low-energy CESM images (R = 0.26, *p* < 0.04) and a high level of correlation for subtraction CESM images (R = 0.67, *p* < 0.01) (Figure 2).

Both pairs of images tended to imprecisely estimate the sizes of residual lesions. In the case of low-energy images, the size of these lesions was overestimated (the average overestimation value was 6.28 mm), whereas in the case of subtraction CESM images, residual lesions were underestimated (the average underestimation value was 2.75 mm).

According to the RECIST 1.1 guidelines, the low-energy images with morphological assessment only revealed 15.87% CR (*n* = 10) and 84.13% non-CR (*n* = 53). In the case of subtraction CESM images, these parameters were 47.62% (*n* = 30) and 52.38% (*n* = 33), respectively. Histopathological examination demonstrated CR in 33.33% (*n* = 21) of cases and non-CRs in 66.67% (*n* = 42). A detailed description of the particular responses to NAC can be found in Table 3.

In the histopathological examination for invasive ductal carcinoma (IDC), a CR of 39.22% (20 out of 51 of tumors) could be achieved, whereas for invasive lobular carcinoma (ILC) it was16.67% (1 out of 6 tumors). In the case of mixed IDC/ILC, CR was not achieved in any of the tumors. In Table 4, differences in the NAC response depending on the type of breast cancer analyzed by CESM and histopathological examination are shown.

Comparing the two types of CESM images to the histopathological examination, Table 5 presents the sensitivity, specificity, negative predictive value (NPV), and positive predictive value (PPV) of both images in the prediction of the CR. The sensitivity of low-energy images in forecasting CR amounted to 33.33%, while its specificity was 92.86%. In the case of the subtraction CESM images, the sensitivity amounted to 85.71% and the specificity to 71.42%.

**Figure 3:**
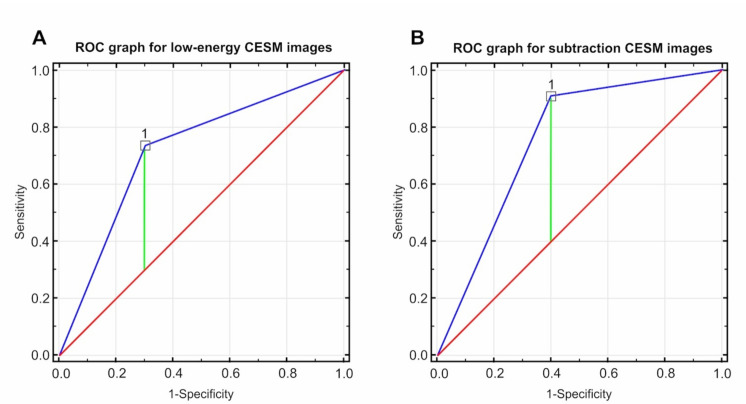
ROC curves based on the tested diagnostic methods (Youden Index:0.44, proposed cut-off point: 1.00): (**A**) for low-energy CESM images, the value of the AUC field was 0.718 at a standard error of 0.091 and *p* < 0.0172; (**B**) for subtraction CESM images, the value of the AUC field was 0.755 at a standard error of 0.064 and *p* < 0.0001.

Figure 3 presents the differences in ROC curves for low-energy and subtraction CESM images in detecting CR.

**Figure 4:**
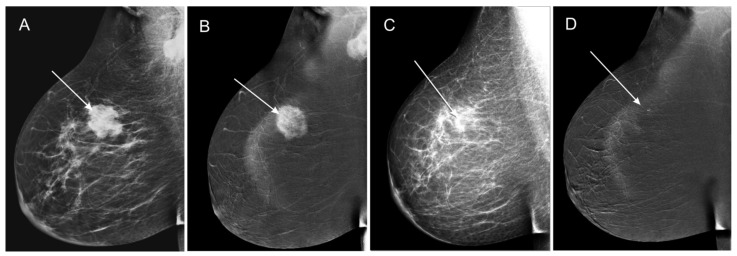
Assessment of therapeutic response in low-energy MLO (**A**,**C**) and subtraction MLO (**B**,**D**). Before NAC, a tumor can be seen in the upper outer quadrant of the right breast with high density and polycyclic outlines, accompanied by enlarged lymph nodes in the axillary fossa (**A**), revealing pathological contrast enhancement in subtraction CESM images (**B**). Following NAC, in the tumor field, there is a visible focal asymmetry, with a density lower than the residual glandular tissue (**C**), without pathological contrast enhancement (**D**). Based on the low-energy images, the therapeutic response was classified as partial response (PR). Based on the subtraction images, the therapeutic response was classified as complete response (CR), which was acknowledged in the HP examination.

**Figure 5:**
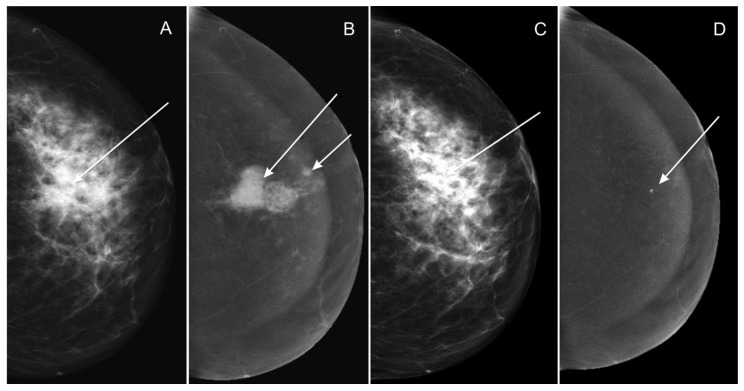
Assessment of therapeutic response in low-energy CESM CC images (**A**,**C**) and subtraction CESM CC images (**B**,**D**) before NAC (IDC LumB, G2 T3N1)showing irregular infiltration on the border of the outer quadrants of the left breast with high density (**A**), revealing pathological contrast enhancement on subtraction CESM images (**B**). Additionally, satellite foci are visible in subtraction CESM images, which were confirmed in core-needle biopsy (smaller arrow) (**B**). Following NAC, there was a visible focal asymmetry, with a density slightly lower than the infiltration before NAC (**C**), shown again without pathological contrast enhancement (**D**). Based on the low-energy CESM images, the therapeutic response was classified as stable disease (SD). Based on the subtraction CESM images, the therapeutic response was classified as complete response (CR), which was acknowledged in the HP examination.

Figure 4 and Figure 5 present an assessment of the therapeutic responses in two pairs of CESM images.

## 4. Discussion

Early assessment of NAC response to breast cancer and correct differentiation between patients with the complete pathological response (pCR) and without NAC response is the key point in NAC therapy. Proper evaluation is crucial to the future clinical perspectives and therapy for each patient. Obtaining complete pathological response results in better event-free survival (EFS) and overall survival (OS) rates [33,34].

In our study, the sensitivity of low-energy images in forecasting CR reached 33.33%, the specificity was 92.86%, the PPV was 70%, and the NPV was73.58%. After the conversion of subtraction images, the sensitivity of CESM in CR detection in a group of patients after NAC was 85.71%, its specificity was 71.42%, the PPV was 60%, and the NPV was 90.90%. Similar results for subtraction CESM images have been acquired by other authors such as Patel et al. [35] (64 patients, sensitivity: 95%, specificity: 66.7%, PPV: 55.9%, NPV: 96.7%), Iotti et al. [36] (46 patients, sensitivity: 100%, specificity: 84%, PPV: 57%, NPV: 100%), and Barra et al. [37] (33 patients, sensitivity: 76%, specificity: 62.5%, PPV: 86%, NPV: 45.4%). All mentioned studies, including ours, showed that, in the assessment of the CR following NAC, subtraction CESM images reached significantly higher sensitivity. Unfortunately, their specificity was lower. These results demonstrate that imaging techniques, even after intravenous administration of a contrast agent, may not differentiate between residual infiltration lesions and co-existing inflammatory/reactive lesions. A similar problem occurs with MRI, which tends to underestimate residual changes [23].

In their meta-analysis, Tang et al. [38] demonstrated that the pooled sensitivity, specificity, positive likelihood ratio (PLR), negative likelihood ratio (NLR), and diagnostic odds ratio (DOR) of the pathological response of breast cancer to NAC assessed by CESM were: 0.83 (95% CI, 0.66–0.93), 0.82 (95% CI, 0.68–0.91), 4.66 (95% CI, 2.59–8.41), 0.20 (95% CI, 0.10–0.43), and 22.91 (95% CI, 8.66–60.62), respectively. The same parameters for MRI, which is considered to be the best method for assessing response to NAC, were as follows: sensitivity: 0.77 (95% CI, 0.67–0.84), specificity: 0.82 (95% CI, 0.73–0.89), PLR: 4.35 (95% CI, 3.00–6.33), NLR: 0.28 (95%CI, 0.20–0.39), and DOR: 15.48 (95% CI, 9.97–24.03). Based on these findings, it can be concluded that CESM has the same specificity and higher sensitivity than MRI and is more accurate in the pathological evaluation of NAC response in breast cancer treatment.

The largest pretreatment tumor dimensions in our analysis of low-energy and subtraction CESM images were similar and there was a statistical difference between these modalities (R = 0.89, *p* = 0.01). However, these differences became significant following neoadjuvant chemotherapy (R = 0.55, *p* = 0.01). This was because post-NAC tumors reduce their density and then become difficult to distinguish from glandular tissue based on morphological images alone. On the other hand, the functional information provided by CESM in subtraction images showed that the residual infiltration was visible, and the type of breast tissue did not affect its visualization. Moreover, in our study, the comparison of the measurement of the maximal sizes of residual changes after NAC, evaluated using CESM subtraction images and histopathology, showed a high correlation (R = 0.67, *p* = 0.01). Iotti et al. [36], while comparing the sizes of tumors after NAC with a histopathological examination, proved that CESM showed greater coherence with histopathology than MRI (Lin’s coefficient were 0.81 and 0.59, respectively). In contrast, Patel et al. [35] achieved the opposite results, where MRI showed higher compatibility with histopathology than CESM (Lin’s concordance coefficient was 0.75 for CESM and 0.76 for MRI; Pearson’s correlation was 0.77 for CESM and 0.80 for MRI). The lack of consistency between researchers indicates the need for further research—multicenter, on a larger group of patients, using equipment available on the market from various companies.

In our study, Low-energy images tended to overestimate the dimensions of residual lesions following NAC, while subtraction CESM images tended to underestimate them. Similar results were acquired by Patel et al. [35] and Iotti et al. [36], where CESM and histopathology results underestimated the size of post-NAC tumors by 5mm and 4.1mm, respectively. It should also be emphasized that the underestimation of the dimensions of residual lesions in our study had no impact on the scope of surgical treatment. Since CESM is a method involving vascularization of the tumor foci, the effect of excessive reduction in vascularization around the tumor during NAC may account for the weaker enhancement of the residual tumor mass on follow-up CESM, underestimating the actual dimension of residual lesions. A similar problem arises with MRI, which tends to underestimate residual lesions in follow-up examinations [35,36]. Over-or underestimation of residual disease can also be a result of the fact that, due to neoadjuvant chemotherapy, induction of cellular changes leading to the elimination of cancerous cells occurs before the decrease in tumor size [39,40]. Moreover, after the eradication of cancerous cells in the region of the tumor, the residual mass visible on radiological images may still be present. It consists of fibrotic tissue left after the therapy. To overcome this problem, Xing et al. [41] and Moustafa et al. [42] suggested relying not only on RECIST 1.1 criteria in the evaluation of NAC response but also creating a special mathematical model. It consists of a combination of the measurement of the largest diameter of the target lesion together with subjective identification of the difference in the intensity of the contrast uptake before and after NAC. After that, a combination of the summation of the number of pixels and their intensity within the area of interest before and after NAC is included in this model. It should be noted that, after using this method, CESM remained a method of high sensitivity and specificity in the evaluation of NAC response. Such results prove that CESM is one of the best methods for diagnostic imaging available for the analysis of the response to neoadjuvant chemotherapy.

The histological and molecular heterogeneity of tumors cannot be evaluated with a simple examination with a contrast medium, and biopsies are limited, especially in large tumors. The need to better define the heterogeneity of tumors will involve the aid of new methodologies currently under study, such as radiomics. Radiomics in MRI can be more effective in the diagnosis of breast cancer and in the histological and morphological assessment of tumors. For CESM, a radiomics model achieved a significantly better discriminative ability compared to the standard clinical model (AUC, 0.81 vs. 0.55, *p* < 0.01) [43,44,45]. In recent years, it has also been suggested to use background parenchymal enhancement (BPE) in the assessment of responses to NAC, which describes the enhancement of the normal breast tissue related to physiological vascularization. La Forgia et al., indicated that BPE is an important aspect that conditions the diagnosis and that it is a potential predictive factor in the response to neoadjuvant cancer therapies in graphic contrast examinations, as already confirmed in MRI and CESM examinations [46,47,48].

CESM, while being a relatively novel diagnostic imaging technique, has become a useful tool in diagnosing and evaluating breast cancer stages. Subtraction images improve the diagnostic specificity of low-energy images, providing more precise measurement of tumor size as well as the possibility of identifying multifocal diseases, especially in women with dense breast tissue. Thus, the effectiveness of CESM in breast cancer diagnosis is comparable to MRI and is a promising tool to serve as a basic imaging technique in patients with symptomatic breast cancer or for the detection of multifocal and multicentric breast cancers [49,50,51]. As we have shown in our study, CESM is also a useful and effective method in assessing the pathological response to NAC. Post-NAC treatment monitoring is extremely important for planning surgical treatment, reducing the number of mutilating mastectomies, and replacing them with breast-conserving surgeries. However, the most accurate measurements possible should be made to avoid underestimating the size of the tumor and increasing the extent of the operation. The advantage of CESM over other methods so far available is its precise definition of the tumor before NAC thanks to the directly integrated visualization of suspicious calcifications in the low-energy images and enhancement in the recombined images, which is not possible with MRI [52].

Our study had several limitations. Firstly, this single-institution research was carried out on a relatively small number of female patients due to the limited number of qualifications for NAC. Moreover, all CESM examinations were conducted on a single vendor system. Finally, we did not include patients with HER2-positive cancer in our study due to the lack of public funding for trastuzumab in this center in the years from2016–2019 (no drug prescription government program). There is a need for multi-institutional studies with larger groups of patients free of the limitations described above in the future.

## 5. Conclusions

Due to the possibility of assessing vascularity, CESM is characterized by high sensitivity in the assessment of CR after NAC. The use of morphological assessment alone is insufficient. CESM correlates well with the sizes of residual lesions from histopathological examination but tends to underestimate the dimensions.

## Figures and Tables

**Table 1:** Patients and tumors characteristics.

Characteristics	Number	Percentage
Number of Patients	63	100%
Age, years (median ± SD)	53.32 ± 9.47	-
Menopause		
Before	27	42.86
After	36	57.14
Molecular characteristics		
LumA	5	8.06
LumB	34	54.84
TNBC1-Jan	24	38.71
Type of tumor		
Mixed IDC/ILC	6	9.50
ILC	6	9.50
IDC	51	80.95
TNM stage upon diagnosis		
T1N+	2	3.17
T2N0	13	20.63
T2N+	13	20.63
T3N0	11	17.46
T3N+	16	25.40
T4N0	3	4.76
T4N+	5	7.94

Abbreviations: IDC—invasive ductal carcinoma; ILC—invasive lobular carcinoma; LumA—luminal A breast cancer; LumB—luminal B breast cancer; TNBC—triple-negative breast cancer; TNM—T, N, and M status.

**Table 2:** Dimensions of the lesions before and after neoadjuvant chemotherapy for low-energy and subtraction CESM images and histopathological examination.

	Minimal Dimension	Maximal Dimension	Mean ± SD
**PL-E CESM (mm)**	15.0	77.0	34.4 ± 12.6
**NL-E CESM (mm)**	0.0	80.0	17.6 ± 15.4
**PS CESM (mm)**	8.0	100.0	34.3 ± 17.4
**NS CESM (mm)**	0.0	66.0	8.5 ± 12.0
**NHP (mm)**	0.0	65.0	11.1 ± 12.8

Abbreviations: PL-E CESM—low-energy CESM images prior to NAC; NL-E CESM—low-energy CESM images after NAC; PS CESM—subtraction CESM images prior to NAC; NS CESM—subtraction CESM images after NAC; NHP—histopathological examination after NAC.

**Table 3:** Individual therapeutic responses to NAC using low-energy and subtraction CESM images.

	Low-Energy Images		Subtraction Images	
	*n*	%	*n*	%
**CR**	10	15.87	30	47.62
**PR**	43	68.25	31	49.20
**SD**	9	14.29	2	3.17
**PD**	1	1.59	0	0

Abbreviations: CR—complete response; PR—partial response; SD—stable disease; PD—progressive disease.

**Table 4:** Differences in NAC response depending on the type of breast cancer.

Pathological Response to NAC	IDC (*n* = 51)			Mixed IDC/ILC (*n* = 6)			ILC (*n* = 6)		
	Low- Energy CESM Images	Subtraction CESM Images	HP	Low-Energy CESM Images	Subtraction CESM Images	HP	Low-Energy CESM Images	Subtraction CESM Images	HP
**CR**	8	25	20	0	2	0	2	3	1
**Non-CR**	43	26	31	6	4	6	4	3	5

Abbreviations: CR—complete response; IDC—invasive ductal carcinoma; ILC—invasive lobular carcinoma; HP—histopathology examination.

**Table 5:** Diagnostic performance indexes for the assessment of complete (CR) and non-complete response (PR, SD, PD) according to RECIST 1.1criteria using low-energy and subtraction CESM images compared to histopathology results.

Assessment	RECIST 1.1	Histopathology CR	Histopathology Non-CR (PR, SD, PD)	
**Low-energy** **CESM images**	**CR**	7	3	PPV: 70.0% 95% CI: 0.35–0.93
	**Non-CR** **(PR, SD, PD)**	14	39	NPV: 73.58% 95% CI: 0.60–0.85
		Sensitivity: 33.33% 95% CI: 0.15–0.57	Specificity: 92.86% 95% CI: 0.81–0.99	
**Subtraction** **CESM images**	**CR**	18	12	PPV: 60.00% 95% CI: 0.41–0.77
	**Non-CR** **(PR, SD, PD)**	3	30	NPV: 90.90% 95% CI: 0.76–0.98
		Sensitivity: 85.71% 95% CI: 0.64–0.97	Specificity: 71.42% 95% CI: 0.55–0.84	

Abbreviations: CR—complete response; PR—partial response; SD—stable disease; PD—progressive disease; PPV—positive predictive value; NPV—negative predictive value.

## Data Availability

Data are available upon special request.
